# A Sporadic Case of a 22-Year-Old Female With Spinal Cord Infarction (SCI) Complicated by SS Pattern Sickle Cell Disease (SCD): A Rare Case Report

**DOI:** 10.7759/cureus.30334

**Published:** 2022-10-15

**Authors:** Gajanan N Umalkar, Gajanan Chavan, Vaishnavi V Kantode, Mayur B Wanjari

**Affiliations:** 1 Department of Emergency Medicine, Jawaharlal Nehru Medical College, Datta Meghe Institute of Medical Sciences (Deemed to be University), Wardha, IND; 2 Department of Medical Surgical Nursing, Smt. Radhikabai Meghe Memorial College of Nursing, Datta Meghe Institute of Medical Sciences (Deemed to be University), Wardha, IND; 3 Department of Research, Jawaharlal Nehru Medical College, Datta Meghe Institute of Medical Sciences (Deemed to be University), Wardha, IND

**Keywords:** magnetic resonance imaging, sickle cell disease, ischemia, myelopathy, spinal cord infarction

## Abstract

A stroke that occurs either in arteries that supply the spinal cord or the spinal cord itself is called spinal cord infarction (SCI). The lower thoracic area is the most typical site for spinal cord infarcts. Spinal infarcts are rare even among people without sickle cell disease, making up only a very less amount of all infarcts to the central nervous system. A 22-year-old female with a known case of SS pattern sickle cell anaemia was brought by her parents to the emergency medicine department with a complaint of pain in the bilateral upper and right lower limbs. The pain progressed to weakness within 15 minutes, which was sudden in onset and associated with faecal and urine incontinence. On physical examination of the client, her Glasgow coma scale (GCS) was E3 VT M5, pupils were bilateral and equally reactive to light, in both upper limbs, the power was 0/5 and 2/5 in the left lower limb, and hypotonia in the upper and right lower limb was noted. Still, the tone was expected in the left lower limb. MRI showed myelopathy extending over three segments from c2 to c4 involving predominantly anterior aspect, most likely cord ischemia. The patient was treated in the neurocritical care unit with tab Ecosprine 150 mg, multivitamins, and rehabilitative therapy. After two months, she showed gradual but consistent improvement in restoring some motor function in her affected limbs. SCIs are uncommon. Although ischemic stroke can be treated with anticoagulants and antiplatelet medicines, viable therapies for SCI have not yet been identified.

## Introduction

Spinal cord infarction (SCI), a severe but unusual condition, is generally less common than ischemic brain injury. One percent to two percent of all neurological and vascular crises are caused by it [1]. Spinal infarcts are rare even among people without sickle cell disease, making up only 1% of all infarcts to the central nervous system [2]. The most frequent neurologic consequence of sickle cell disease (SCD) is cerebral infarction; it can be severe or asymptomatic and is associated with serious morbidity [3]. Patients with SCD may get cytotoxic medications and erythrocyte transfusions as part of their treatment, including timely physical therapy and care for underlying causes [4]. The origin of a spinal infarct is frequently unknown [5].

## Case presentation

A 22-year-old female with a known case of SS pattern sickle cell anaemia was brought by her parents to the emergency medicine department with a complaint of pain in the bilateral upper and right lower limbs. The pain progressed to weakness within 15 minutes, which was sudden in onset and associated with faecal and urine incontinence. Her parents brought her in a drowsy state. On arrival, the Glasgow coma scale (GCS) was 13/15. But later, the airway was threatened with 75 % saturation on room air because of bradypnea and decreased respiratory effort; she was immediately intubated for abnormal breathing. She was in the red category as per the triage early warning score (TEWS). She has been a known case of SS pattern sickle cell anaemia for the last five years, and she is continuously on tab hydroxyurea 500 mg twice a day and tab folic acid.

On physical examination of the client, her GCS was E3 VT M5, pupils were bilateral and equally reactive to light, in both upper limbs the power was 0/5 and 2/5 in the left lower limb, and hypotonia in the upper and right lower limbs was noted. Still, the tone was normal in the left lower limb. On admission, a blood test was done; the level of haemoglobin was 8 gm%. Peripheral smear showed microcytic hypochromic with mild anisopoikilocytosis anaemia. Cultures of the blood and urine came negative.

**Figure 1:**
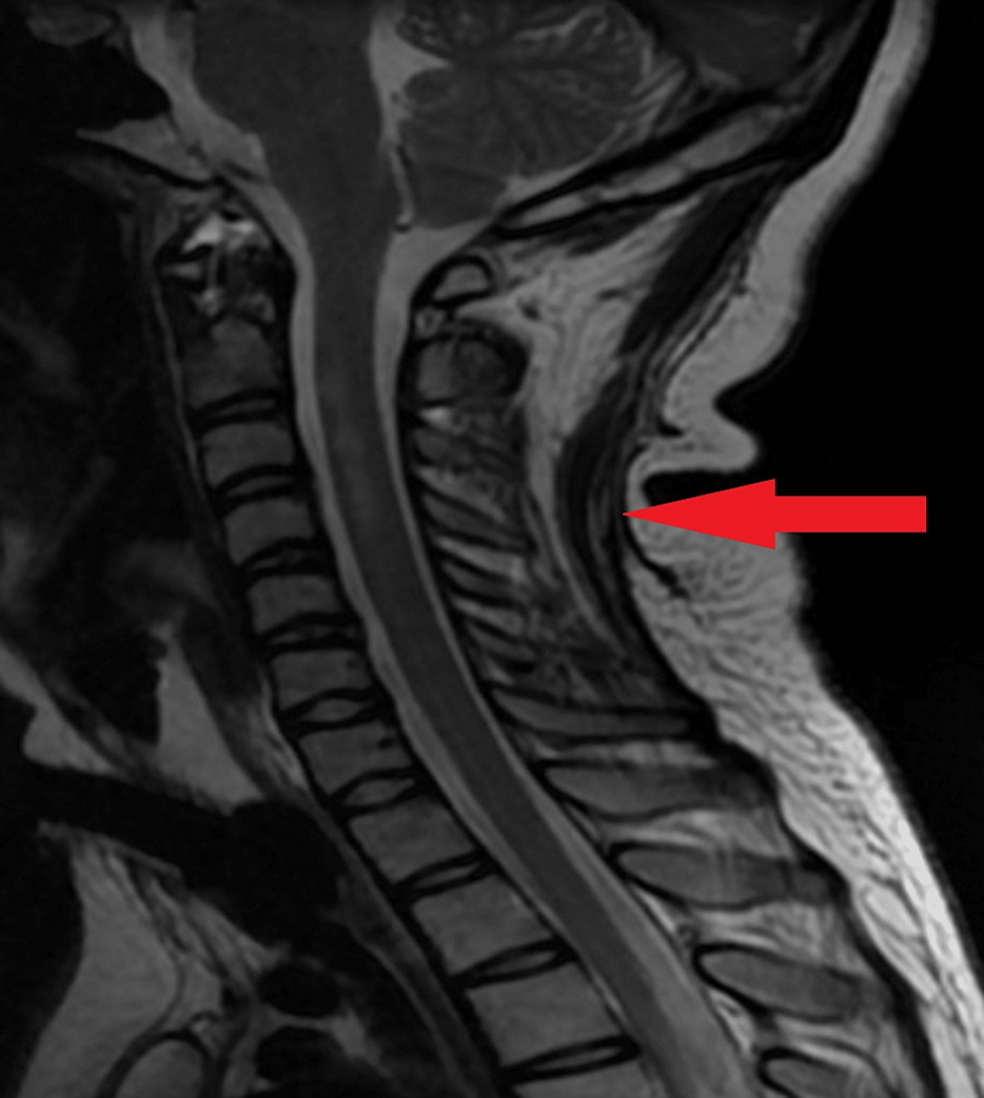
Shows myelopathy extending over three segments from c2 to c4 involving predominantly anterior aspect

The level of erythrocyte sedimentation rate (ESR) and C-reactive protein (CRP) were both elevated. There were no abnormalities seen on the brain's CT scan, and then the patient was advised for an MRI of the cervical spine; the whole spine screening was suggestive of myelopathy extending over three segments from c2 to c4 involving predominantly anterior aspect, most likely cord ischemia (Figure1).

She was shifted to the neurocritical care unit (NCCU), where the patient was treated with a tab of aspirin 150 mg once a day, multivitamins, and adequate nutrition and hydration provided through Ryles tube and intravenous, respectively, and later she was tracheostomized. She was discharged from NCCU after 19 days and treated with nursing care and rehabilitative therapy only. Two months after, the patient's tracheostomy tube was removed. She showed gradual but consistent improvement in restoring some motor function in her affected limbs.

## Discussion

The most frequent SCI risk factors include atherosclerosis, hypertension, diabetes, and cardioembolism [6]. Spinal cord infarcts are often more frequent in younger individuals than cerebral infarcts and have a slight female predominance in patients [7]. Although SCI is a rare disorder, it can have many clinical presentations and severe neurological side effects such as paraplegia, quadriplegia, and incontinence. Clinically, SCI might be distinguished from other nontraumatic myelopathy aetiologies by three key symptoms: hyperacute temporal course, new-onset back pain, and flaccid weakness [8]. The above-mentioned clinical symptoms and findings from MRI, such as focal cord swelling and pencillike hyperintensities on T2-weighted images, are required to diagnose SCI [9].

The clinical presentation can range from moderate weakness to tetraplegia depending on the affected vascular region. It might be challenging to diagnose by an emergency room doctor [10]. Myelopathy has been infrequently characterized, with few published cases of compressive and ischemic myelopathy, even though neurological problems in sickle cell patients are widely known [11]. An MRI is helpful to find out other acute myelopathy causes, such as transverse myelitis, demyelination lesions, tumour constriction of the cord, spondylosis, and disc herniations [12].

In contrast, ischemic stroke can be managed with antiplatelet medication and thrombolysis, but effective treatments for SCI have not yet been developed. Conventional rehabilitation is the primary treatment method for paraplegia, spasticity, and sensory disruption. The primary strategy for assisting people in their efforts to achieve independent walking is traditional therapy alone because there is no efficient medical treatment for SCI [13]. There are no established guidelines for treating SCI because of the unique nature of the disorder and the variety of aetiologies and symptoms involved. [14]. Different outcomes can be expected depending on the severity of the SCI and the subsequent recovery. Brain infarction generally has a worse long-term prognosis than SCI [15].

## Conclusions

SCIs are uncommon. The lower thoracic cord is where they are most likely to occur. Although many spinal cord infarcts are unexplained, the aortic disease is the most frequently found aetiology. Even though the first clinical manifestation of SCI is diverse and nonspecific, it should be considered when weakness and sensory abnormalities appear suddenly. Early SCI diagnosis is essential, and a spine MRI with diffusion-weighted imaging (DWI) should be performed as soon as feasible to provide proper therapy. From this case, we have concluded that the cause for the sudden onset of paraplegia or quadriplegia should not be only cerebral infarction and degenerative myelin disorder. We should also suspect other reasons, as we found in this case, SCI; early diagnosis and early treatment could improve the patient's life.
